# Antibody-Dependent Neutrophil Phagocytosis of *Plasmodium falciparum*–Infected Erythrocytes Is Mediated by FcγRIIa

**DOI:** 10.1093/infdis/jiag071

**Published:** 2026-02-05

**Authors:** Maria Saeed, Elizabeth H Aitken, Bruce D Wines, Stephen J Rogerson

**Affiliations:** Department of Infectious Diseases; Department of Infectious Diseases; Department of Microbiology and Immunology, The Peter Doherty Institute for Infection and Immunity, University of Melbourne; Immune Therapies Group, Burnet Institute for Medical Research and Public Health; Department of Immunology, School of Translational Medicine, Monash University; Department of Infectious Diseases; Department of Medicine, Peter Doherty Institute for Infection and Immunity, University of Melbourne, Australia

**Keywords:** ADNP, FcγRIIa, FcγRIIIb, malaria, phagocytosis

## Abstract

**Background:**

Fc gamma receptor III b (FcγRIIIb), a glycosylphosphatidylinositol-linked receptor, is the most abundant neutrophil FcγR on neutrophils, followed by FcγRIIa. FcγRs interact with IgG, and studies have reported the association of antibody-dependent neutrophil phagocytosis (ADNP) with protection against malaria; however, the role of specific FcγRs is not clear.

**Methods:**

To investigate the relative importance of FcγRIIIb and FcγRIIa as mediators of the ADNP of *Plasmodium falciparum*–infected erythrocytes, purified neutrophils from healthy donors were treated with tumor necrosis factor (TNF) to mobilize the intracellular stores of FcγRIIIb to the surface, followed by enzymatic cleavage of glycosylphosphatidylinositol-linked FcγRIIIb with phosphatidylinositol phospholipase C (PIPLC).

**Results:**

In TNF/PIPLC-treated neutrophils, detectable FcγRIII decreased by 79% (relative geometric mean fluorescence intensity, 21 ± 4.5 [mean ± SD]), while FcγRIIa detection increased by 82% (182 ± 2.3) as compared with untreated neutrophils (100%). When opsonized infected erythrocytes were incubated with TNF/PIPLC-treated neutrophils, ADNP by FcγRIIIb-depleted neutrophils increased significantly (relative phagocytosis, 585% ± 108%) as compared with untreated neutrophils (100%, *P* = .042). Using FcγR blocking, we show that when compared with no blocker (relative phagocytosis, 100%), ADNP was reduced >5-fold by FcγRIIa blocker alone (∼17% ± 1.5%, *P* < .05) and to a similar extent by combined FcγRIIa and FcγRIII blockers (∼24% ± 5.5%, *P* < .05).

**Conclusions:**

Our data suggest that FcγRIIa is the main phagocytic receptor that mediates the ADNP of infected erythrocytes and that FcγRIIIb acts as a decoy receptor.

## Teaser

We investigated the Fc gamma receptors (FcγRs) that neutrophils use to phagocytose antibody-opsonized *Plasmodium falciparum*–infected red blood cells. Following tumor necrosis factor stimulation and enzyme treatment, FcγRIIIb expression decreased, but phagocytosis increased. Blocking FcγRIIa significantly decreased phagocytosis. FcγRIIa, not FcγRIIIb, mediates antibody-dependent neutrophil phagocytosis.

Malaria continues to be a global health concern, causing 263 million cases and 597 000 deaths in 2023 [1]. With antimalarial drug and insecticide resistance on the rise, there is a need, more than ever, to develop effective vaccines for malaria [1]. The World Health Organization, together with funding associates, aims to develop a ≥75% efficacious vaccine by 2030 [2]. To achieve this goal, there is a need to better understand the mechanisms driving the protective immune responses to malaria.

One reason why *Plasmodium falciparum* is the deadliest among the *Plasmodium* species is that infected erythrocytes (IEs) sequester in the vasculature. Sequestration is largely mediated by expression of *P falciparum* erythrocyte membrane protein 1 (PfEMP1), a family of large multidomain proteins on the surface of IEs, which mediate cytoadhesion of IEs to a range of host cell receptors [3]. During pregnancy, parasites switch to express the VAR2CSA PfEMP1, which binds to chondroitin sulfate A on the placental syncytiotrophoblast [4], resulting in placental sequestration [5]. Switches in PfEMP1 expression help the parasite avoid recognition and clearance by the host immune system [6].

Preparations of immunoglobulins from malaria-exposed individuals have been shown to reduce parasitemia, establishing a key role of antibodies in protection [7]. Seroepidemiologic studies suggest that cytophilic subclasses of IgG (IgG1 and IgG3) are the most important in protection [8, 9]. One of the most important functions of IgG1 and IgG3 is to engage Fc gamma receptors (FcγRs) on immune cells, which leads to the release of cytokines and inflammatory mediators and/or opsonic phagocytosis [10, 11].

FcγRs are present on immune cells, including neutrophils, the most abundant leukocytes. Antibody-dependent neutrophil phagocytosis (ADNP) is initiated by engagement of neutrophil FcγRs with the Fc region of IgG in immune complexes. It is important to understand the specific FcγRs mediating ADNP, since this process results in the uptake and destruction of many IgG-opsonized targets, including bacteria [12], viruses [13], and tumors [14]. For *P falciparum*, while ADNP has been described for sporozoites [15, 16], merozoites [16, 17], and whole IEs [18], the specific FcγRs mediating the ADNP of IEs have not been identified. Understanding this process would inform the development of antibody-based therapeutics and vaccines.

Neutrophils express high levels of FcγRIIIb (CD16b), at 100 000 to 200 000 copies per cell; moderate levels of FcγRIIa (CD32a), at 30 000 to 60 000 copies; and little to no FcγRIIIa (CD16a). FcγRI is absent but can be induced by interferon gamma [19]. FcγRIIIb is a glycosylphosphatidylinositol (GPI)–anchored receptor that lacks transmembrane and cytoplasmic signaling domains [20]. FcγRIIIb is postulated to be involved in functions such as Ca^2+^-dependent actin polymerization [21–23], NETosis [24], and β1 integrin activation [25], but despite its abundance it has not been shown to participate directly in phagocytosis of IgG-coated beads and bacteria [26–28]. In addition to the surface FcγRIIIb, neutrophils have plentiful intracellular reserves of FcγRIIIb [29, 30], while FcγRIIa does not have an intracellular pool [27]. Also, in contrast to FcγRIIIb, FcγRIIa has a cytoplasmic domain bearing an immunoreceptor tyrosine-based activating motif [20] essential for mediating phagocytosis [27]. Further signaling differences are that FcγRIIa and FcγRIIIb mediate distinct PKC isoform signaling pathways [23, 31].

Studies that have investigated the role of neutrophil FcγRs in phagocytosis of *P falciparum* merozoites and sporozoites have used FcγR blocking antibodies and reported FcγRIIIb to be the main receptor [15, 17]. Notably, these antibodies cannot differentiate between the highly homologous extracellular domains of FcγRIIIa and FcγRIIIb [32]. Studies of neutrophil phagocytosis of IgG-opsonized beads or *Staphylococcus aureus*, by contrast, indicated that FcγRIIa is the main receptor [27, 28, 33]. Since these studies with different targets of phagocytosis revealed varied dominance of FcγRs, the objective of this study was to identify the key FcγRs that mediate the ADNP of IEs. We adopted an approach that circumvented any possible difficulty of blocking the highly abundant FcγRIIIb by specifically cleaving FcγRIIIb from the surface of neutrophils using the phosphatidylinositol phospholipase C (PIPLC) enzyme. Furthermore, neutrophil activation mobilized intracellular FcγRIIIb to the cell surface for cleavage, and so the role of FcγRs in the ADNP of IEs was rigorously addressed. We show that removal of FcγRIIIb increases, rather than decreases, the ADNP of IEs whereas subsequent blocking of FcγRIIa significantly decreases ADNP. This observation confirms that FcγRIIa is the major receptor involved in the antibody-dependent phagocytosis of IEs by tumor necrosis factor (TNF)–treated and untreated neutrophils.

## METHODS

### Parasite Culture and Purification of *P falciparum* Trophozoites

A *P falciparum* CS2 line was cultured in the laboratory as previously described [34]. Sorbitol lysis was used to synchronize the cultures [35], and the gelatin floatation method was used for the selection of knobby parasites expressing VAR2CSA-type PfEMP1 [36]. MycoAlert kit (Lonza) was used to confirm that the cultures were mycoplasma negative, per the manufacturer's instructions. For the phagocytosis assay, mature trophozoite-stage IEs were purified via Percoll gradient to at least 95% purity, stained with dihydroethidium (25 μg/mL; Sigma-Aldrich) for 30 minutes in the dark at room temperature, washed, and resuspended in RPMI-HEPES at 4.2 × 10^7^ IEs/mL.

### Preparation of Neutrophils

Written informed consent for blood collection was obtained from healthy Melbourne donors who were malaria naive. Neutrophils were purified from whole blood with an EasySep Direct Human Neutrophil Isolation Kit (STEMCELL Technologies), following the manufacturer's instructions. Cells were counted with a hemocytometer, and viability was checked by trypan blue staining. Polymorphonuclear cell purity was investigated by light microscopy after staining with Rapid-Diff (Australia Biostain) for 1 minute. Neutrophil purity (>95%) was confirmed by flow cytometry with anti-human CD66b-BV421 antibody (Clone G10F5, 1:400; BD Bioscience; Supplementary Figure 1).

### ADNP of *P falciparum* IEs

To assess the ADNP of CS2 IEs, we used 3 μL of positive controls (polyclonal rabbit anti-human erythrocyte antibody, RaH; Cappel). Positive controls comprised pooled plasma samples (PPSs) collected at 16 to 28 weeks’ gestation from 27 Malawian women with high levels of antibody to CS2 IEs and/or high levels of THP-1–mediated phagocytosis of IEs, who were participating in a malaria prevention trial [37]. Negative controls consisted of nonimmune plasma from healthy Melbourne participants and serum-free RPMI-HEPES. Controls were incubated with 12 μL of dihydroethidium-stained IEs (4.2 × 10^7^ IEs/mL in RPMI-HEPES) in a 96-well U-bottom plate precoated with 0.1% bovine serum albumin for 1 hour in the dark at room temperature. After opsonization, IEs were washed thrice to remove unbound antibodies and resuspended in 50 μL of neutrophil medium (RPMI-1640 supplemented with 10% fetal bovine serum and 1% penicillin-streptomycin-glutamine). In a separate 96-well U-bottom plate, 50 μL of neutrophils (5 × 10^5^ cells/mL) were plated per well, and 25 μL of opsonized IEs were added to each well. The plate was incubated for 1 hour at 37 °C with 5% CO_2_ for phagocytosis. After incubation, the plate was centrifuged at 4 °C at 350*g* for 5 minutes, and any nonphagocytosed uninfected erythrocytes or IEs were lysed with 1× lysing solution (BD Biosciences). Cells were washed and fixed with 2% paraformaldehyde in phosphate-buffered saline for 15 minutes and resuspended in 100 μL of FACS buffer. Plates were stored in 4 °C overnight before acquisition on a flow cytometer (CytoFLEX S; Beckman Coulter). Neutrophils were gated by forward scatter and side scatter parameters, and ADNP was measured by gating on dihydroethidium-positive neutrophils (Supplementary Figure 1e).

### Phosphatidylinositol PIPLC Treatment of Neutrophils to Cleave GPI-Linked FcγRIIIb

Neutrophils (50 μL) at 1 × 10^7^ cells/mL were incubated with 25 ng/mL of TNF (R&D Systems) for 15 minutes at 37 °C with 5% CO_2_ with gentle agitation. After priming, neutrophils were incubated in the absence (control) or presence of the PIPLC enzyme (Thermo Fisher Scientific) at 0.96 U/mL for 30 minutes at 37 °C in 5% CO_2_ with gentle agitation. In a separate well, cells were stained with anti-human CD59-FITC (BD Biosciences), a GPI-linked cell surface marker, to confirm the efficiency of PIPLC treatment. After enzymatic treatment, neutrophils were resuspended in 50 μL of neutrophil medium, and 25 μL of opsonized IEs were added to the neutrophils (as previously described) to examine the effect of TNF with and without PIPLC treatment on the ADNP of IEs.

### Blocking FcγRs

Untreated neutrophils and neutrophils treated with TNF and PIPLC were incubated with FcγR blockers (ie, anti-human FcγRIIa; monoclonal antibody [mAb] IV-3) [38] or a combination of anti-human FcγRIIa and anti-human FcγRIII (clone 3G8; BD Pharmingen) for 30 minutes at 4 °C in the dark. After blockage of FcγRs, the unbound blocking antibodies were washed away or kept during phagocytosis. Next, the opsonized IEs were added to neutrophils, and ADNP was measured as described previously. Phagocytosis was quantified by flow cytometry, and analysis was performed in FlowJo software (version 10). Unopsonized IEs were included as a negative control in the phagocytosis assays.

### Data Analysis

FcγRIII and FcγRIIa expression was presented as the geometric mean fluorescence intensity (gMFI) relative to untreated control. For ADNP by untreated neutrophils, data were presented as percentage phagocytosis. To assess the effect of TNF and PIPLC treatment on the ADNP of IEs, data were presented as percentage phagocytosis relative to untreated neutrophils incubated with RaH-opsonized IEs. For FcγR blocking experiments, data were presented relative to untreated neutrophils incubated with RaH-opsonized IEs, without blockers.

Statistical analysis was conducted in Prism version 10 (GraphPad Software Inc). Statistical tests and *P* values are provided in the figure legends. For regular phagocytosis assay, an unpaired *t* test was performed. Group-wise comparisons were done by paired *t* test. Statistical significance was defined as *P* < .05.

## RESULTS

### ADNP of *P falciparum* IEs

**Figure 1.**
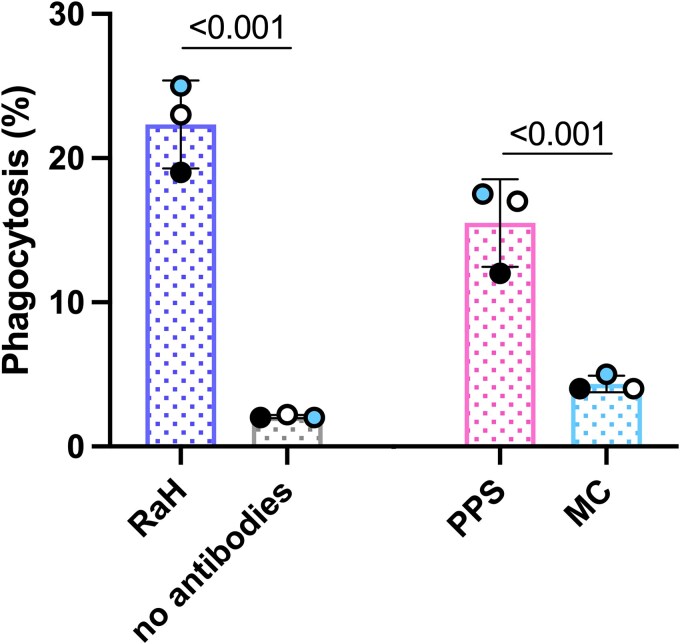
Phagocytosis of *Plasmodium falciparum*–infected erythrocytes (IEs) by neutrophils. Opsonized IEs were incubated with purified neutrophils for 1 hour at 37 °C, and the phagocytosis of IEs was measured in the presence of polyclonal rabbit anti-human erythrocyte IgG (RaH), immune pooled plasma (PPS), nonimmune plasma from Melbourne controls (MC), and no opsonin (no antibodies). Graphs represent percentage of neutrophils that phagocytosed at least 1 IE. Results (mean ± SD, n = 3) are from 3 experiments run in duplicate. Individual experiments are color coded. Comparisons are by unpaired *t* test.

The ADNP of CS2 IEs was assessed with purified neutrophils from healthy Melbourne donors who were malaria naive. The results are shown as percentage of neutrophils that phagocytosed ≥1 IEs. The proportions of neutrophils ingesting IEs opsonized with RaH (mean ± SD, 22% ± 1.8%) and PPS (17% ± 0.76%) were significantly higher than unopsonized IEs (2.1% ± 0.07%, *P* = .0003) and Melbourne controls (4.3% ± 0.33%, *P* = .0002), respectively (Figure 1). This shows that antibody-dependent phagocytosis can be assessed by the assay.

### PIPLC Cleaves FcγRIIIb From the Surface of Neutrophils

**Figure 2.**
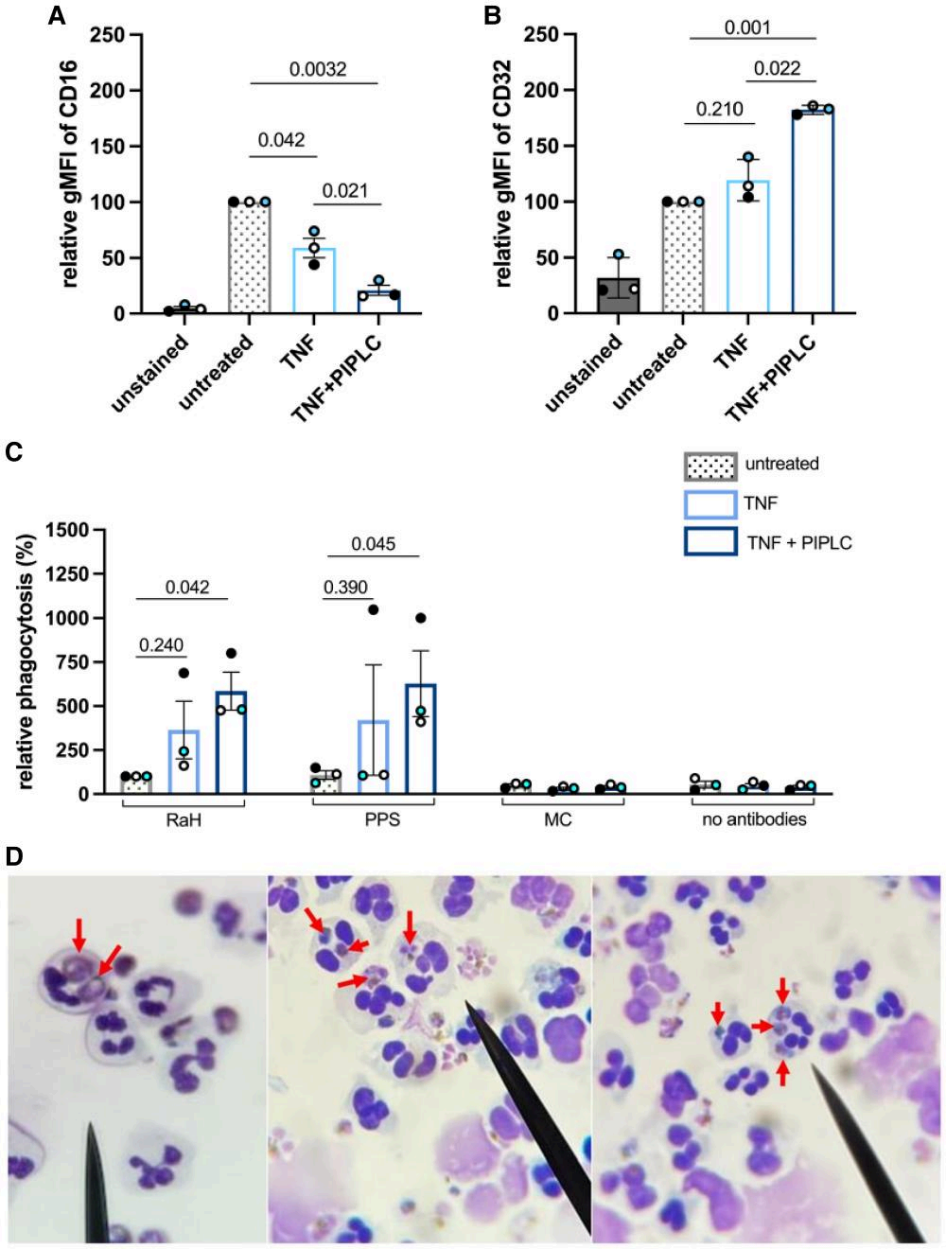
Effect of tumor necrosis factor (TNF) and phosphatidylinositol phospholipase C (PIPLC) treatment on neutrophil Fc gamma receptor III b (FcγRIIIb) and FcγRIIa expression and opsonic phagocytosis of infected erythrocytes (IEs). Neutrophils were untreated (control) or treated with TNF (25 ng/mL) or TNF and PIPLC (100 U/mL) and then stained with anti-CD16 PE-Cy7 and anti-CD32 APC for 15 minutes. *A* and *B*, Geometric mean fluorescence intensity (gMFI) of CD16 and CD32 relative to that of untreated neutrophils. *C*, FcγRIIIb removal by TNF + PIPLC treatment increases the neutrophil phagocytosis of IEs opsonized with rabbit anti-human (RaH) erythrocyte antibody and pooled sera from individuals exposed to malaria (pooled plasma sample [PPS]). Also shown are IEs opsonized by nonimmune plasma from Melbourne controls (MC) and serum-free RPMI-HEPES (no sera). Percentage phagocytosis is shown relative to that of untreated neutrophils incubated with RaH-opsonized IEs. Results (mean ± SD, n = 3) are from 3 experiments run in duplicate. Individual experiments are color coded. Comparisons are by paired *t* test. *D*, Light microscopy images show opsonized IEs (red arrows) internalized by neutrophils.

Neutrophils were briefly treated with TNF to mobilize the intracellular FcγRIIIb to the surface and treated with PIPLC to cleave FcγRIIIb. The efficiency of PIPLC treatment to cleave GPI-anchored receptors was confirmed by the near complete removal of CD59, another GPI-linked protein (Supplementary Figure 2). Combined TNF/PIPLC treatment resulted in a 79% decrease in FcγRIII levels (relative gMFI, 21 ± 4.5) as compared with untreated neutrophils (100%, *P* = .0032) and a 65% decrease vs neutrophils treated with TNF alone (59 ± 8.6, *P* = .021). Notably, TNF treatment alone did not increase the gMFI of FcγRIII; instead, there was a decreased gMFI of FcγRIII as compared with untreated neutrophils (*P* = .042; Figure 2*A*). By contrast, the gMFI of FcγRIIa was increased in combined TNF/PIPLC-treated neutrophils (relative gMFI, 182 ± 2.3) vs untreated neutrophils (100%, *P* = .001) or neutrophils treated with TNF alone (relative gMFI, 119 ± 10, *P* = .022). The brief treatment with TNF alone did not result in a significant increase in the gMFI of FcγRIIa as compared with untreated neutrophils (*P* = .21; Figure 2*B*).

### Removal of FcγRIIIb Increases the ADNP of *P falciparum* IEs

To determine the role of FcγRIIIb in the ADNP of IEs, we first cleaved FcγRIIIb from the cell surface and then used neutrophils in phagocytosis assays. It was observed that the ADNP of RaH-opsonized IEs by FcγRIIIb-depleted neutrophils was nearly 6-fold higher (relative phagocytosis, 585% ± 108%) when compared with untreated neutrophils (100%, *P* = .042; Figure 2*C*). Likewise, neutrophils treated only with TNF, which showed partial FcγRIIIb depletion (Figure 2*A*), were nearly 4-fold more active in the ADNP of RaH-opsonized IEs (relative phagocytosis, 366% ± 164%) than the untreated neutophils (*P* = .240). Similar results were observed when FcγRIIIb-depleted neutrophils were incubated with PPS-opsonized IEs (628% ± 187%) as compared with untreated neutrophils incubated with PPS-opsonized IEs (108% ± 25%, *P* = .045). Light microscopy images show that IEs were internalized by neutrophils (Figure 2*D*), and cytochalasin D treatment caused significant impairment of phagocytosis (Supplementary Figure 3).

### Blocking FcγRIIa Decreases the ADNP of *P falciparum* IEs

**Figure 3.**
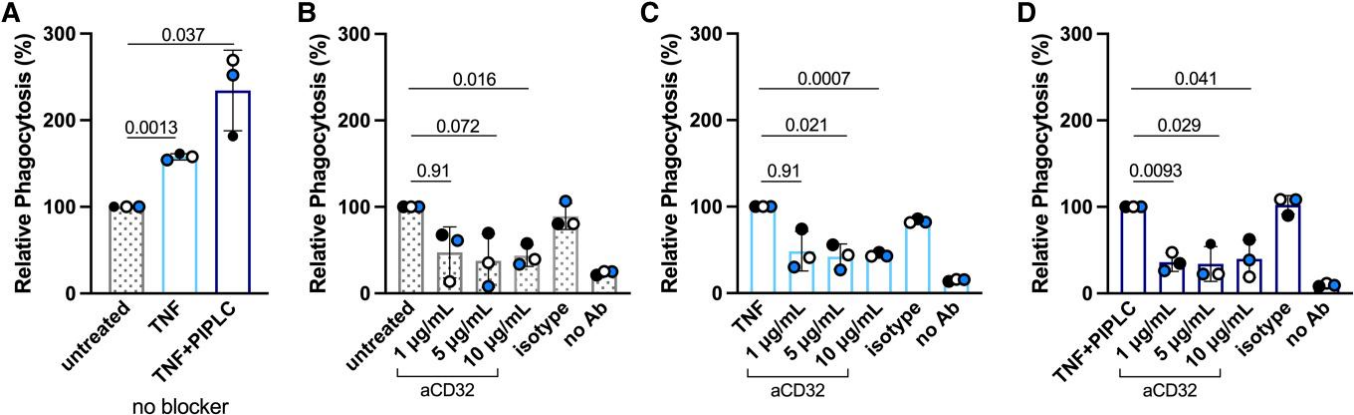
Effect of blocking antibody to FcγRIIa on the phagocytosis of IEs by TNF- and PIPLC-treated neutrophils. Neutrophils untreated, treated with TNF, or treated with TNF and PIPLC and incubated with the FcγRIIa blocking mAb IV-3 or an isotype control for 30 minutes. The unbound mAb was removed, and the neutrophil preparations were incubated with RaH-opsonized IEs. *A*, Percentage phagocytosis is shown relative to that of untreated neutrophils in the absence of any blocking mAb. *B–D*, Results are shown as percentage phagocytosis relative to that of untreated neutrophils, TNF-treated neutrophils, and TNF + PIPLC–treated neutrophils, each in the absence of any blocking mAb. Results (mean ± SD, n = 3) are from 3 experiments run in duplicate. Individual experiments are color coded. Comparisons are by paired *t* test. Abbreviations: Ab, antibody; FcγRIIa, Fc gamma receptor II a; IE, infected erythrocyte; mAb, monoclonal antibody; PIPLC, phosphatidylinositol phospholipase C; RAH, rabbit anti-human; TNF, tumor necrosis factor.

Because the phagocytosis of opsonized IEs increased after removal of FcγRIIIb from neutrophils (Figure 2*C*), we hypothesized that FcγRIIa is mediating the phagocytosis. Therefore, we treated neutrophils with TNF and PIPLC, blocked FcγRIIa, washed out the FcγRIIa-blocking mAb, and determined its effect on ADNP (Figure 3). Untreated neutrophils, TNF-treated neutrophils, and TNF/PIPLC-treated neutrophils were incubated with RaH-opsonized IEs in the absence of any blocker (Figure 3*A*). TNF-treated neutrophils showed greater phagocytosis (relative phagocytosis, 158% ± 2.1%; *P* = .0013), and phagocytosis by TNF/PIPLC-treated neutrophils was even higher (234% ± 27%, *P* = .037) relative to untreated neutrophils (100%). Despite the washing away of FcγRIIa blocker after incubation and before the addition of opsonized IEs, FcγRIIa blocking resulted in a partial reduction in ADNP in all 3 preparations of neutrophils (Figure 3*B*–*D*), with the effects of blocking most apparent for the combined TNF/PIPLC-treated neutrophils. Phagocytosis by these TNF/PIPLC-treated neutrophils was reduced by the FcγRIIa blocking mAb at 1 μg/mL (relative phagocytosis, 36% ± 6.2%), 5 μg/mL (34% ± 12%), and 10 μg/mL (40% ± 13%) as compared with no blocker (100%; *P* = .0093, .029, and .041 respectively; Figure 3*D*).

**Figure 4.**
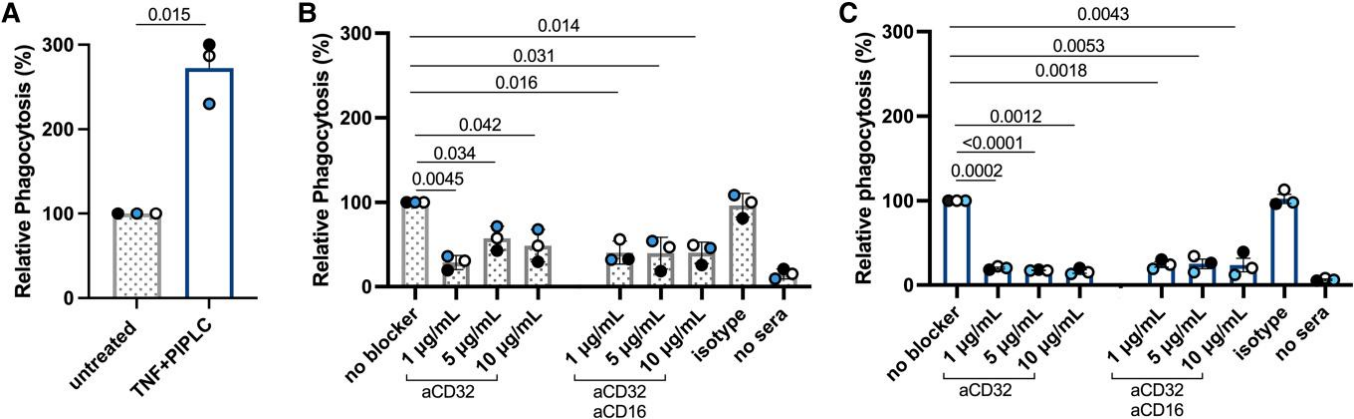
Effect of FcγRIIa blocking mAb and combined FcγRIIa and FcγRIII blocking on the phagocytosis of infected erythrocytes by untreated and TNF- and PIPLC-treated neutrophils. *A*, The neutrophils, untreated or treated with TNF/PIPLC, were incubated with no blocker as controls. *B* and *C*, The neutrophils, untreated or treated with TNF/PIPLC, were incubated with FcγRIIa blocking mAb IV-3 (aCD32) or a combination of mAb IV-3 and 3G8 (aCD16), specific for FcγRIII, for 30 minutes. The blocking mAbs were present during phagocytosis. Percentage phagocytosis is shown relative to that of untreated and TNF- and PIPLC-treated neutrophils in the absence of any blocking mAb. Results (mean ± SD, n = 3) are from 3 experiments run in duplicate. Individual experiments are color coded. Comparisons are by paired *t* test. Abbreviations: FcγRIIa, Fc gamma receptor II a; FcγRIII, Fc gamma receptor III; mAb, monoclonal antibody; PIPLC, phosphatidylinositol phospholipase C; TNF, tumor necrosis factor.

The assays were repeated with anti-FcγRIIa (IV-3) alone and combined with anti-FcγRIII (3G8; Figure 4). Untreated and TNF/PIPLC-treated neutrophils were incubated with RaH-opsonized IEs in the absence of any blocker (Figure 4*A*). Relative to untreated control, phagocytosis by FcγRIIIb-depleted neutrophils was significantly higher (relative phagocytosis, 272% ± 21%) as compared with untreated control (100%, *P* = .015).

In the next experiments, the blocking mAbs were present during phagocytosis to ensure that the Fcγ receptors remained blocked during phagocytosis. Relative to untreated neutrophils in the absence of any blocking mAb (Figure 4*B*), FcγRIIa blocking of untreated neutrophils significantly reduced phagocytosis at 1 μg/mL (29% ± 4.8%), 5 μg/mL (57% ± 8.2%), and 10 μg/mL (48% ± 11%) as compared with untreated cells (100%; *P* = .0045, .034, and .042, respectively). Combining FcγRIIa and FcγRIII blocking mAb did not further reduce ADNP beyond FcγRIIa blocking alone. FcγRIII blocking mAb alone caused a small decrease in phagocytosis (Supplementary Figure 4).

Similarly, FcγRIIa blocking of TNF/PIPLC-treated neutrophils significantly reduced phagocytosis at 1 μg/mL (20% ± 1.2%), 5 μg/mL (17% ± 0.33%), and 10 μg/mL (16% ± 2.9%) as compared with no blocker (TNF/PIPLC cells, 100%; *P* = .0002, <.0001, and .0012, respectively; Figure 4*C*). Combining FcγRIIa and FcγRIII blocking mAb did not further reduce ADNP beyond FcγRIIa blocking alone at at 1 μg/mL (24% ± 3.2%), 5 μg/mL (25% ± 5.5%), and 10 μg/mL (24% ± 8.0%) as compared with no blocker (TNF/PIPLC cells, 100%; *P* = .0018, .0053, and .0043).

## DISCUSSION

Antibody clearance of *P falciparum* asexual blood-stage parasites is key to resolution of malaria infection. Our group previously reported the ADNP of *P falciparum* IEs as a correlate of protection from placental malaria [18]. ADNP is also critical in the clearance of other *P falciparum* stages. Studies have reported that the ADNP of *P falciparum* merozoites is associated with protection from febrile malaria [16, 17] and that the ADNP of *P falciparum* sporozoites has a role in clearing them from peripheral blood [15]. Although researchers have explored the role of ADNP in malaria immunity, studies investigating the key FcγRs involved in ADNP in malaria are limited.

Herein we demonstrate a critical role for FcγRIIa in neutrophil-mediated phagocytosis of opsonized *P falciparum* IEs. The more abundant GPI-linked FcγRIIIb resides on the neutrophil surface and in secretory granules [29, 30]. Brief treatment with TNF mobilized the intracellular receptor to the cell surface, and PIPLC was used to specifically cleave FcγRIIIb from these neutrophils, leaving FcγRIIa as the major FcγR. After cleavage of FcγRIIIb, FcγRIIa was more readily detectable on the neutrophil surface, and phagocytosis of *P falciparum* IEs markedly increased, highlighting the functional importance and inducible activity of this receptor. Specific blocking of FcγRIIa with mAb IV-3 confirmed the predominate role of FcγRIIa in the phagocytosis of *P falciparum* IEs by these treated neutrophils.

Some features of the treatment of neutrophils with TNF and PIPLC are noteworthy. When neutrophils are treated with TNF alone, it mobilizes the intracellular FcγRIIIb to the surface, and in doing so the neutrophil membrane undergoes actin polymerization, resulting in activation-induced shedding of FcγRIIIb, hence the reduction in FcγRIIIb observed [29, 30, 39]. Interestingly, minor residual FcγRIII expression was observed on the TNF/PIPLC-treated neutrophils, possibly from incomplete liberation of FcγRIIIb and/or a contribution by FcγRIIIa, which can be expressed at low levels on neutrophils [26]. FcγRIIIa is a transmembrane receptor and so is not removed by PIPLC treatment, nor can it be distinguished from FcγRIIIb by available detecting antibodies due to the near identity of the 2 receptors’ extracellular domains. The expression and role of FcγRIIIa on neutrophils form an important question, and this TNF/PIPLC treatment may allow further clarification of its role, which has thus far been defined by using neutrophils from an individual who is FcγRIIIb deficient [26]. There was no difference in FcγRIIa detection after TNF treatment, as reported previously [40, 41]. However, after TNF/PIPLC treatment, we observed increased detection of FcγRIIa, which could be due to possible TNF-induced activation and clustering of FcγRIIa over short incubation times [41] or to increased accessibility to detecting antibodies after the removal of FcγRIIIb and other GPI-linked receptors.

The improved IE phagocytosis activity of the TNF/PIPLC-treated neutrophils that lack FcγRIIIb may result from antibody-opsonized IEs being better able to engage with FcγRIIa. Removing the near 10-fold excess of FcγRIIIb over FcγRIIa must focus the opsonized IE interaction on the FcγRIIa receptor. Selvaraj et al reported that coexpression of FcγRIIIb may serve to limit the ligand-binding role of FcγRIIa [42]. Indeed, it has been reported that FcγRIIIb acts as a decoy receptor for IgG-opsonized targets and that ADCC of cancer cells by neutrophils is completely FcγRIIa dependent [43]. Added to this, brief TNF treatment alters FcγRIIa behavior, increasing binding to IgG-opsonized ligands without an increase in expression levels [41, 44]. The increased FcγRIIa binding to IgG-opsonized ligands may result from the loss of FcγRIIIb and/or the effect of TNF treatment on the binding properties of FcγRIIa [41, 42].

To clarify the role of FcγRIIa in phagocytosis, we blocked FcγRIIa alone or in combination with FcγRIII blocking on PIPLC-treated neutrophils, which removed most FcγRIIIb, and we left the blockers in the solution to try to ensure blocking during phagocytosis. We observed marked reduction in the ADNP of IEs following FcγRIIa blocking. Combining FcγRIIa and FcγRIII blockers did not reduce ADNP beyond the effect of FcγRIIa blocking alone. Overall, our results are consistent with the previous evidence that FcγRIIa is the major neutrophil phagocytic receptor, as demonstrated variously in the ADNP of IgG-coated latex beads [27, 33] and that of IgG-opsonized *S aureus* [28]. It is worth noting that these results contrast with studies of the ADNP of merozoites and sporozoites that showed FcγRIIIb as the main receptor with FcγRIIa synergy [15, 17], which could be due to TNF priming of the neutrophils.

Our studies are of clinical importance since TNF is produced during *P falciparum* infection and recruits and activates neutrophils. Some studies show that high levels of TNF are sufficient to control parasitemia and prevent symptoms [45, 46], while a meta-analysis showed that TNF levels are higher in patients with severe malaria as compared with uncomplicated malaria [47]. We show that FcγRIIa is the key mediator in the ADNP of *P falciparum* IEs in TNF-activated neutrophils in vitro, suggesting that the protective role of TNF seen in vivo [45, 46] may, at least in part, be mediated through its enhancing action on the phagocytic activity of FcγRIIa.

A limitation of this study is that the expression of FcγRI before and after TNF treatment was not measured. However, marked inhibition of phagocytosis after FcγRIIa blocking suggests that even if FcγRI was induced, it is unlikely to have played a major role in the phagocytosis. Furthermore, there is evidence that IFN-γ or granulocyte colony-stimulating factor and not TNF can, with relatively prolonged incubation, upregulate FcγRI expression on neutrophils [19, 48]. Other GPI-anchored receptors on neutrophils include CD24, CD48, GPI-80, CD59, and CD157. How FcγR-mediated phagocytosis may be affected upon shedding of these molecules from the neutrophils is, to the best of our knowledge, not known.

This study establishes the importance of IgG Fc-FcγR engagement on neutrophils for the phagocytosis of malaria IEs and widens the context of protection by this antibody-dependent neutrophil function from previous studies of bacteria pathogens [49] and viruses, including HIV [13] and Ebola [50]. This infomation could also be employed in malaria vaccine development to induce antibodies with high affinity to FcγRIIa to promote ADNP.

In summary, our study shows that FcγRIIa is the main phagocytic receptor involved in the ADNP of *P falciparum* IEs and blocking FcγRIIa significantly reduces ADNP.

## Supplementary Material

jiag071_Supplementary_Data

## Supplementary Data

Supplementary materials are available at *The Journal of Infectious Diseases* online (http://jid.oxfordjournals.org/). Supplementary materials consist of data provided by the author that are published to benefit the reader. The posted materials are not copyedited. The contents of all supplementary data are the sole responsibility of the authors. Questions or messages regarding errors should be addressed to the author.
