# Supplementary material for: Antibody-Dependent Neutrophil Phagocytosis of Plasmodium falciparum–Infected Erythrocytes Is Mediated by FcγRIIa
Source: J Infect Dis. 2026 Feb 5;233(4):e882–90. doi: 10.1093/infdis/jiag071 (PMC13127751; doi:10.1093/infdis/jiag071)
Supplement: jiag071_Supplementary_Data [file jiag071_supplementary_data.docx]

**Supplementary Figures**


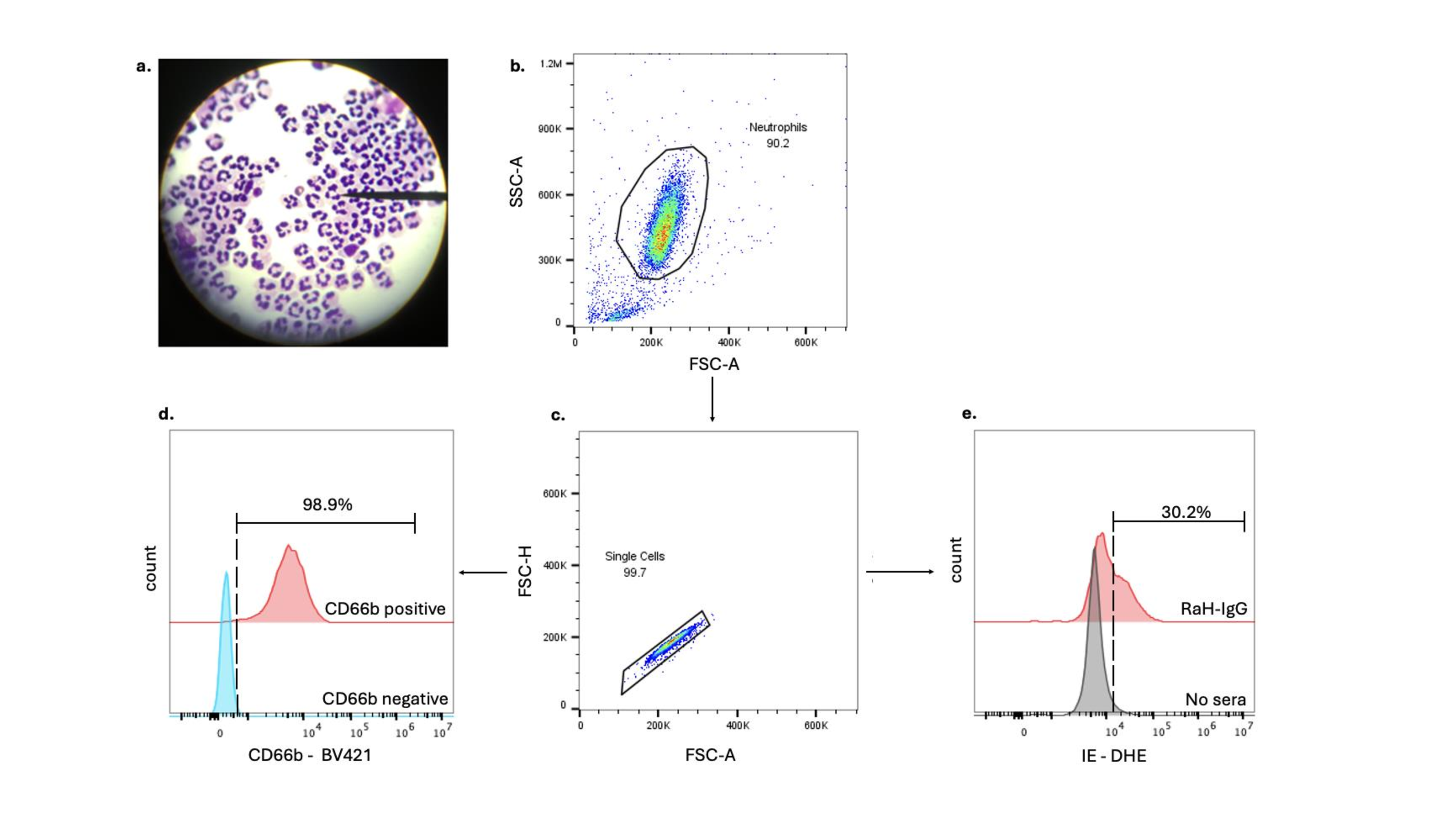


**Supplementary Figure S1. Neutrophil purity and gating strategy. (a)** Isolated neutrophils were visualized by microscopy after cytospin (Rapid Diff 1 and 2 stained). **(b)** Neutrophils were gated based on forward scatter (FSC-A) vs. side scatter (SSC-A). **(c)** Singlets were gated based on forward scatter area (FSC-A) vs height (FSC-H). **(d)** Neutrophil purity was confirmed by gating on CD66b-positve events. **(e)** Opsonic phagocytosis was measured by gating on DHE-positive events and excluding non-opsonic phagocytosis.


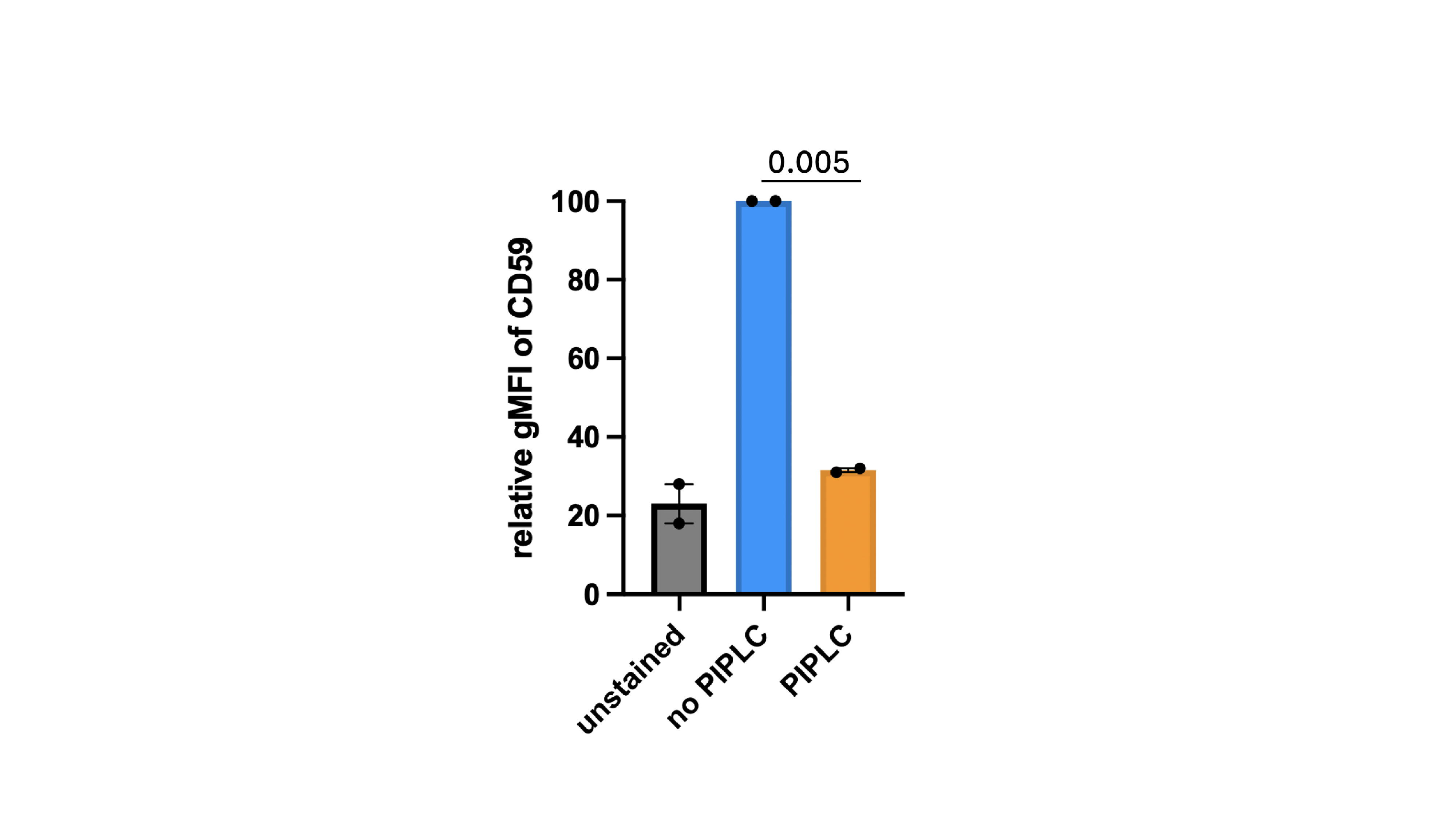


**Supplementary Figure S2. PIPLC (phosphatidylinositol phospholipase C) cleaves GPI (glycosylphosphatidylinositol) anchored receptor CD59 from neutrophils.** Neutrophils were incubated with 0.96 U/mL PIPLC for 30 minutes at 37ºC with gentle agitation. CD59 expression was measured by flow cytometry. Graph shows the expression of CD59 presented as gMFI (geometric mean fluorescence intensity) relative to untreated control (no PIPLC). Results (mean ± standard deviaiton, n= 2) are of two experiments, run in duplicate. Comparisons are by paired t-test.


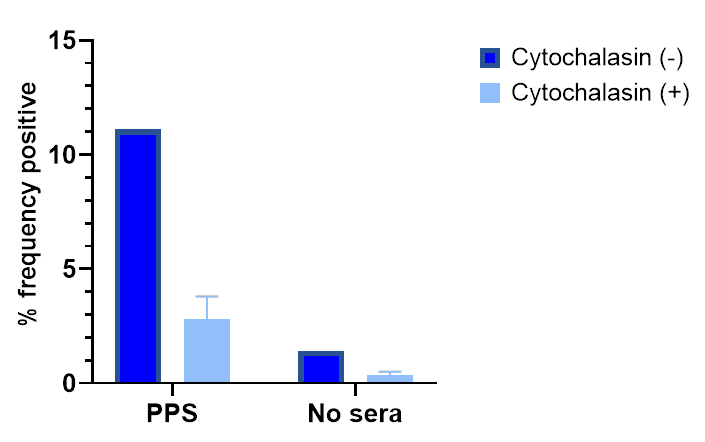


**Supplementary Figure S3. Effect of cytochalasin D treatment on phagocytosis.** Unstimulated neutrophils were treated with cytochalasin D (Sigma) at 5 µM for 15 min at room temperature, washed once, and incubated with pooled positive plasma (PPS) opsonised IE or unopsonised IE (No sera). Single experiment performed in duplicate.


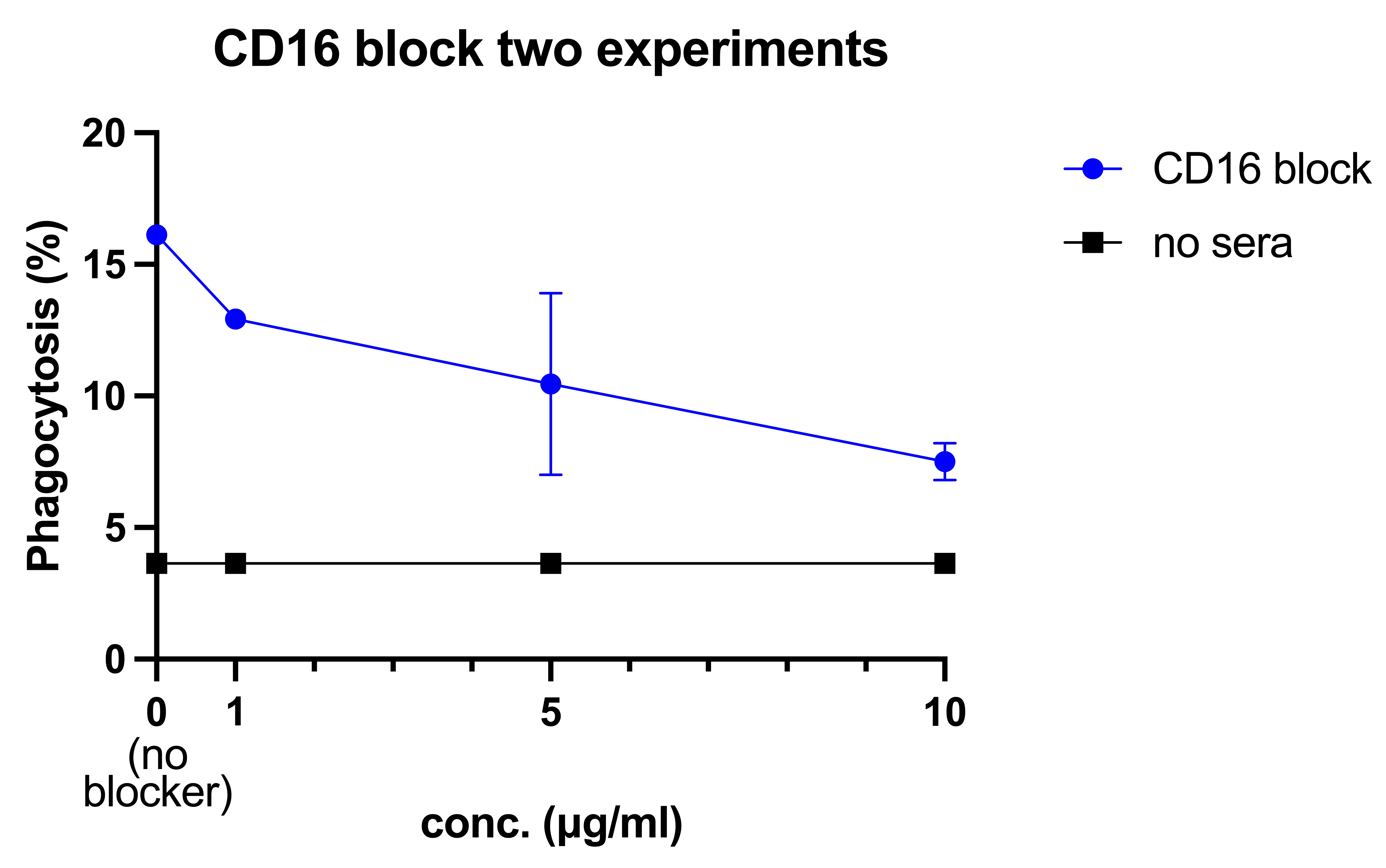


**Supplementary Figure S4. Effect of CD16 blocking monoclonal antibody on phagocytosis of IEs by untreated neutrophils.** The untreated neutrophils were incubated without CD16 blocker (0 μg/mL) and CD16 blocker at 1 μg/mL, 5 μg/mL and 10 μg/mL for 30 minutes. The blocking mAbs were present during phagocytosis. Graph shows percentage phagocytosis by neutrophils. Results are presented as average of two experiments run in duplicates.
